# Spread of Epidemic MRSA-ST5-IV Clone Encoding PVL as a Major Cause of Community Onset Staphylococcal Infections in Argentinean Children

**DOI:** 10.1371/journal.pone.0030487

**Published:** 2012-01-23

**Authors:** Claudia Sola, Hugo Paganini, Ana L. Egea, Alejandro J. Moyano, Analia Garnero, Ines Kevric, Catalina Culasso, Ana Vindel, Study Group of CA-MRSA in Children, Argentina-2007, Horacio Lopardo, José L. Bocco

**Affiliations:** 1 Centro de Investigaciones en Bioquímica Clínica e Inmunología (CIBICI-CONICET), Departamento de Bioquímica Clínica; Facultad de Ciencias Químicas, Universidad Nacional de Córdoba, Córdoba, Argentina; 2 Hospital de Pediatría “Prof. Dr.Juan P. Garrahan”, Autonomous city of Buenos Aires (ACBA), Argentina; 3 Hospital de Niños de la Santísima Trinidad de Córdoba, Córdoba, Argentina; 4 Laboratorio de Infecciones Nosocomiales, Instituto de Salud Carlos III, Centro Nacional de Microbiología, Majadahonda, Madrid, Spain; Universite Libre de Bruxelles, Belgium

## Abstract

**Background:**

Community-associated methicillin-resistant *Staphylococcus aureus*-(CA-MRSA) strains have emerged in Argentina. We investigated the clinical and molecular evolution of community-onset MRSA infections (CO-MRSA) in children of Córdoba, Argentina, 2005–2008. Additionally, data from 2007 were compared with the epidemiology of these infections in other regions of the country.

**Methodology/Principal Findings:**

Two datasets were used: *i)* lab-based prospective surveillance of CA-MRSA isolates from 3 Córdoba pediatric hospitals-(CBAH1-H3) in 2007–2008 (compared to previously published data of 2005) and *ii)* a sampling of CO-MRSA from a study involving both, healthcare-associated community-onset-(HACO) infections in children with risk-factors for healthcare-associated infections-(HRFs), and CA-MRSA infections in patients without HRFs detected in multiple centers of Argentina in 2007. Molecular typing was performed on the CA-MRSA-(n: 99) isolates from the CBAH1-H3-dataset and on the HACO-MRSA-(n: 51) and CA-MRSA-(n: 213) isolates from other regions. Between 2005–2008, the annual proportion of CA-MRSA/CA-*S. aureus* in Córdoba hospitals increased from 25% to 49%, *P*<0.01. Total CA-MRSA infections increased 3.6 fold-(5.1 to 18.6 cases/100,000 annual-visits, *P*<0.0001), associated with an important increase of invasive CA-MRSA infections-(8.5 fold). In all regions analyzed, a single genotype prevailed in both CA-MRSA (82%) and HACO-MRSA(57%), which showed pulsed-field-gel electrophoresis-(PFGE)-type-“I”, sequence-type-5-(ST5), SCC*mec*-type-IVa, *spa*-t311, and was positive for PVL. The second clone, pulsotype-N/ST30/CC30/SCC*mec*IVc/t019/PVL^+^, accounted for 11.5% of total CA-MRSA infections. Importantly, the first 4 isolates of Argentina belonging to South American-USA300 clone-(USA300/ST8/CC8/SCC*mec*IVc/t008/PVL^+^/ACME^−^) were detected. We also demonstrated that a HA-MRSA clone-(pulsotype-C/ST100/CC5) caused 2% and 10% of CA-MRSA and HACO-MRSA infections respectively and was associated with a SCC*mec* type closely related to SCC*mec*IV(2B&5).

**Conclusions/Significance:**

The dissemination of epidemic MRSA clone, ST5-IV-PVL^+^ was the main cause of increasing staphylococcal community-onset infections in Argentinean children (2003–2008), conversely to other countries. The predominance of this clone, which has capacity to express the h-VISA phenotype, in healthcare-associated community-onset cases suggests that it has infiltrated into hospital-settings.

## Introduction

Methicillin resistant *Staphylococcus aureus* (MRSA) infection has traditionally been associated with healthcare settings (HA-MRSA) until its emergence in the community during late 1990's. This global phenomenon was caused by strains of community-associated methicillin-resistant *Staphylococcus aureus* (CA-MRSA) that emerged from some major genetic lineages, commonly designated by their multilocus sequence type (MLST) or by their pulsed field gel electrophoresis (PFGE) pattern (ST1, ST8-USA300, ST30, ST59, ST93 and ST80),with a specific geographical pattern. These strains are frequently non-multiresistant to antibiotics; most of them harbor the staphylococcal cassette chromosome *mec* (SCC*mec*) type IV(2B) or V(5C2) and the Panton-Valentine leukocidin (PVL) genes [1]–[3]. Purulent skin and soft tissue infections are the most common clinical manifestations of CA-MRSA, although increasing reports of serious infections in children with high mortality rates have raised concern during recent years [4], [5]. Vancomycin has been the mainstay of treatment for this type of infections and the emergence of resistance is worrying [6], [7]. Importantly, CA-MRSA strains have entered the healthcare settings; hence, in some countries, strains of both, CA-MRSA and HA-MRSA circulate in hospitals and communities, causing infections of healthcare or community onset (HO and CO, respectively) [8]–[10].

In Latin America, three PVL-positive epidemic CA-MRSA clones have been described: *i)* MRSA ST30-IV clone [11], *ii)* MRSA ST5-IV clone [12], [13] and *iii)* MRSA ST8-IV clone [14]. The first two were detected in southern areas of South America and the third one in the North.

Epidemiological surveillance for MRSA in Córdoba, Argentina, revealed the emergence of ST5-IV-PVL^+^ genotype as the most prevalent (89%) CA-MRSA clone in 2005. This clone had displaced in the community setting an “escaped” HA-MRSA clone, which was non-multiresistant to antibiotics, lacked the *pvl* genes and was characterized as ST100 associated with a SCC*mec* related to IVc [12], [15]. Prospective surveillance of community onset *S. aureus* infections in children from Argentina during 2007, detected a prevalence of 62% (281/447 CA-*S. aureus*) of CA-MRSA infections; 38% of which were invasive infections [16].

A paucity of data about molecular epidemiology of CA-MRSA infections from South America is a current problem and therefore, it is difficult to follow evolving trends [17]–[19].

Herein, we report the molecular evolution and antimicrobial resistance profiles of MRSA strains in the community along with the clinical characteristics of these infections in children of Córdoba-Argentina between 2003 and 2008.

We also compared the clinical and molecular epidemiology of CO-MRSA infections in children with risk factors for healthcare-associated infections (HRFs), (healthcare-associated, community-onset infections [HACO]), and in patients without HRFs (CA-MRSA infections), from multiple centers of central, eastern and northern regions of Argentina during 2007.

## Materials and Methods

### Ethics statement

This study was reviewed and approved by the institutional Ethical Review Board of each Hospital: Hospital de Pediatría Prof. Dr. Juan P. Garrahan; Hospital General de Niños Dr. Pedro de Elizalde; Hospital de Niños Dr. O. Alassia, Santa Fe; Hospital de Niños de San Justo; Hospital Juan Pablo II, Corrientes; Hospital de Niños J. Vilela, Santa Fe; Hospital Pediátrico Dr. A.L. Castelán, Chaco; Hospital Materno Infantil de Mar del Plata Don Victorio Tetamanti, Hospital de Niños de la Santísima Trinidad de Córdoba, Hospital Infantil Municipal de Córdoba; Hospital Pediátrico del Niño Jesús de Córdoba. Written informed consent was obtained from each child's parents for review of the medical information and characterizations of the responsible isolates. However, for the aims of this study, data were processed anonymously. Additionally, the authors declare that all clinical investigation has been conducted according to the principles expressed in the Declaration of Helsinki.

### Study design and case definitions

#### Longitudinal study, Córdoba, 2003–2008

To analyze the molecular evolution and clinical characteristics of CA-MRSA infections in children (≤18 years of age) in Córdoba, all consecutive single patient isolates (n: 99) identified as CA-MRSA according to CDC criteria (see definitions) by a laboratory-based surveillance program of *S. aureus* infections during 2007–2008 from three children's hospitals (CBAH1, CBAH2 and CBAH3) were prospectively included.

These molecular data were compared with those obtained in 2005 [12]. In that year, all *S. aureus* infections were identified by the Clinical Microbiology Laboratory of each hospital (CBAH1, CBAH2 and CBAH3) and the medical records were reviewed for epidemiologic classification (see definitions).

Additionally, 22 CA-MRSA isolates prospectively collected in CBAH1 between 2003 and 2006 following the CDC criteria (see definition) were analysed as well, along with those detected for this study (2007–2008).

#### Transversal study, Argentina, 2007

To examine the clinical and molecular epidemiology of CA-MRSA and HACO-MRSA infections among children from central, northern and eastern regions of Argentina in 2007, random samples representative of each hospital participating in the prospective surveillance of community onset *S. aureus* infections in children from Argentina (CSACHARG) were analyzed. The clinical features, frequency and outcome of CA-MRSA infections identified in that study have already been published [16]. Briefly, a total of 840 *S. aureus* infections were diagnosed during 2007, 582 of them were of community onset; among them, 135 children with HRFs were excluded from that study (75 infected with HACO-MRSA and 60 with HACO-MSSA). Of the remaining 447 isolates, 281 (62%) were CA-MRSA and 160 CA-MSSA. The following sample was selected for this study: CA-MRSA isolates: 213 of 281 (Buenos Aires city: 101, Buenos Aires province: 11, Corrientes: 22, Chaco: 19 and Santa Fe: 60). HACO-MRSA isolates: 51 of 75 (Buenos Aires city: 25, Buenos Aires province: 5, Corrientes: 5, Chaco: 5 and Santa Fe: 11).

For each case of *S. aureus* isolated from community onset infections from both collections (CBAH1-H3 and CSACHARG), the relevant medical information was examined, including diagnosis, site and clinical details of infection, treatment information, demographic data (age, gender), underlying illnesses (chronic conditions) [8], [20] and the presence of the following risk factors for healthcare-associated infections: existence of an invasive device (e.g., vascular catheter, G-tube); any history of MRSA infection or colonization, surgery, hospitalization, dialysis, or residence in a long-term care facility within the 12 months prior to the culture [21]–[23].

### Definitions

Community-associated methicillin resistant *S. aureus* (CA-MRSA) infections were defined as cases from patients without HRFs during the previous year, who acquired the infection outside hospital settings or within 48 h of admission, according to the CDC criteria [22].

Healthcare-associated infections (HA-MRSA) were defined using previously published definitions [21], [22], which include: i) the classical nosocomial or hospital-onset infection (HO-MRSA), defined as a those occurring >48 h after hospital admission, not present on admission (no cases of this type were analyzed in this study) and ii) Healthcare-associated community-onset (HACO-MRSA) infections: those occurring in patients with HRFs, having a positive culture within ≤48 h after hospital admission. Hence, Community-onset methicillin resistant *S. aureus* (CO-MRSA) infections include both the CA-MRSA and HACO-MRSA infections.

Invasive infections were defined by one or more of the following conditions: bacteremia, endocarditis, pneumonia, pleural abscess, lymphadenitis, septic arthritis, osteomyelitis, necrotizing fasciitis, pyomyositis or another illness in which *S. aureus* was isolated from normally sterile body fluids [20], [21]. Sepsis was defined on the basis of the systemic inflammatory response syndrome criteria [24]. Skin diseases (skin and soft tissue, SSTI) were defined as a primary skin infection such as an abscess, cellulitis, folliculitis or a skin infection spreading to contiguous tissues.

Surgical site infections (SSI) were not considered as skin diseases.

### Bacterial isolates and antimicrobial susceptibility

*Staphylococcus aureus* were identified by standard microbiologic procedures. Antimicrobial susceptibility testing was performed by disk diffusion method [25]. A disc approximation test (D test) was used to detect inducible clindamycin resistance [25]. Screening for reduced susceptibility to vancomycin (VISA and h-VISA) was performed on all CO-MRSA isolates. Vancomycin and teicoplanin minimum inhibitory concentrations (MICs) were determined by agar dilution method [25]. In addition, all MRSA isolates with a MIC of 1 or 2 µg/ml were further screened by 2 additional agar methods: MHA5T-Mueller-Hinton-agar and BHI4V-brain-heart-infusion-agar (5 µg/ml teicoplanin and 4 µg/ml vancomycin, respectively) [6], [26], [27]. MRSA isolates that were positive in at least one screening test were additionally tested by both, the Etest Macromethod (MET) and Etest glycopeptide-resistance detection strip-GRD E-test strips, (bioMérieux-CD) [6], [28]. The strains h-VISA-Mu3 and VISA-Mu50 and the vancomycin-susceptible *S. aureus* strain ATCC 29213 were tested for all methods in parallel as positive and negative controls, respectively.

### Molecular typing and detection of genes associated with virulence

In all CO-MRSA isolates, the PFGE of *Sma*I digests of chromosomal DNA were performed and interpreted as previously described [12]. *SmaI* restriction patterns (PFGE) were digitized, presented schematically and analyzed to calculate the Dice coefficient of correlation and to generate a dendrogram by the unweighted-pair group method using average linkage clusterings. Isolates differing by up to six fragments were considered to be subtypes of a given clonal type, and the similarity cut-off was 80%.

All CO-MRSA isolates were screened by PCR for accessory gene regulator (*agr*) type and for 22 specific staphylococcal virulence genes, as described elsewhere [12 and reference therein], comprising sequences specific for enterotoxins: *sea*, *seb*, *sec*, *sed*, *see*, *seg*, *seh*, *sei*, *sej*, *sen*, *seo*, *sem* and *sek*; toxic shock syndrome toxin 1(TSST-1): *tst*; exfoliative toxins: *eta* and *etb*; PVL genes: *lukS*-PV-*lukF*-PV; leukocidin: *lukE-lukD* and the class F leukocidin: *lukM*; bacteriocine (*bsa*), adhesins: for collagen (*cna*) and for bone sialoprotein-binding protein (*bbp*). Detection of *arcA* gene, an indicator of the arginine catabolic mobile element (ACME), was analyzed with primers designed in this study, based on the previously determined sequence of strain USA300_FPR3757 (accession NC_007793). The primer sequences are as follows: *arc*A-F 5′-TCT ATT ACT GAG CCA GAA GTA CG-3′and *arc*A-R 5′-CAC GTA ACT TGC TAG AAC GAG TA-3′, whose product has 733 bp expected size. USA300-0114 was used as reference strain for the USA300 clone.

Representative isolates of the most prevalent subtype (defined by PFGE) from all CO-MRSA strains were also characterized by multilocus sequence typing and *spa* typing [29], [30].

### SCC*mec* typing

The SCC*mec* types (I–VI) were evaluated for all CO-MRSA isolates by multiplex PCR [31] and by allotyping by conventional PCR [32]. SCC*mec* type IV was further sub-typed using published primers [33]. Furthermore, the assignment to the class B *mec* complex was confirmed by PCR with primers specific for ψIS*1272* and *mec*R1 [34]and in order to differentiate the types of SCC*mec* IV and VI, the *ccrAB* typing was completed by PCR detection with primers specific for *ccrAB* allotype 4 [34].

To investigate the presence of SCC*mec* IV(2B&5) [35], all isolates belonging to pulsotype C/ST100 associated with a new variant of SCC*mec* IV (IVNv), which was positive by PCR for J1 specific region of the SCC*mec* subtype IVc and *ccrAB*2 but negative for B *mec* complex [15], were further characterized for the *mecA* downstream vicinity with primers designed based on the sequence previously determined of strain ZH47-(ST100), accession AM292304, (File S1).

The primer sequences are as follows (location relative to the ZH47 sequence given in parentheses): **i)** Tn*4001*-1-F, 5′-GCC AAT CGC TTA ATT GGA GCC G-3′ (18.146 to 18.167) and Tn*4001*-1-R, 5′-ACT TCA TCT TCC CAA GGC TCT GT-3′ (18.854 to 18.832); amplifying the region IS1256L-*aac*(2′)—*aph*(6″) within the Tn*4001*. **ii)** Tn*4001*-2-F, 5′-TGG CCA TCA CGT GTT CTG GG-3′ (21.164 to 21.183) and Tn*4001*-2-R, 5′-TCG GAT GTC TGT CCG AGG ACT-3′ (22.131 to 22.111); amplifying the region between the IS256R (Tn*4001*) and IS1272 from *mec* complex B and **iii)** Tn*4001*-3-F, 5′-ACC AAA CCC GAC AAC TAC AAC TAT-3′ (15.976 to 15.999) and Tn*4001*-3-R, 5′-GTG TCG TAA AGC TGC GCT CA-3′ (17.273 to 17.2554); amplifying the region between the *mec*A locus and IS256L (Tn*4001*). The amplicons with the expected size were end sequenced and compared to the SCC*mec* IV(2B&5) sequence, in the ZH47 strain-(ST100) [35]. All primers used for SCC*mec* IVNv characterization are shown in detail in (File S1).

### Statistical analysis

Bacteriologic and patient data were compiled in an electronic database using Access (Microsoft). Data were analyzed with Epi Info version 6.0.4 software (Centers for Disease Control and Prevention, Atlanta, GA). Comparisons between groups were performed with chi-square test or Fisher's exact test, as appropriate. *P*<0.05 was considered statistically significant.

## Results

### Temporal trends in CA-MRSA infections

**Figure 1:**
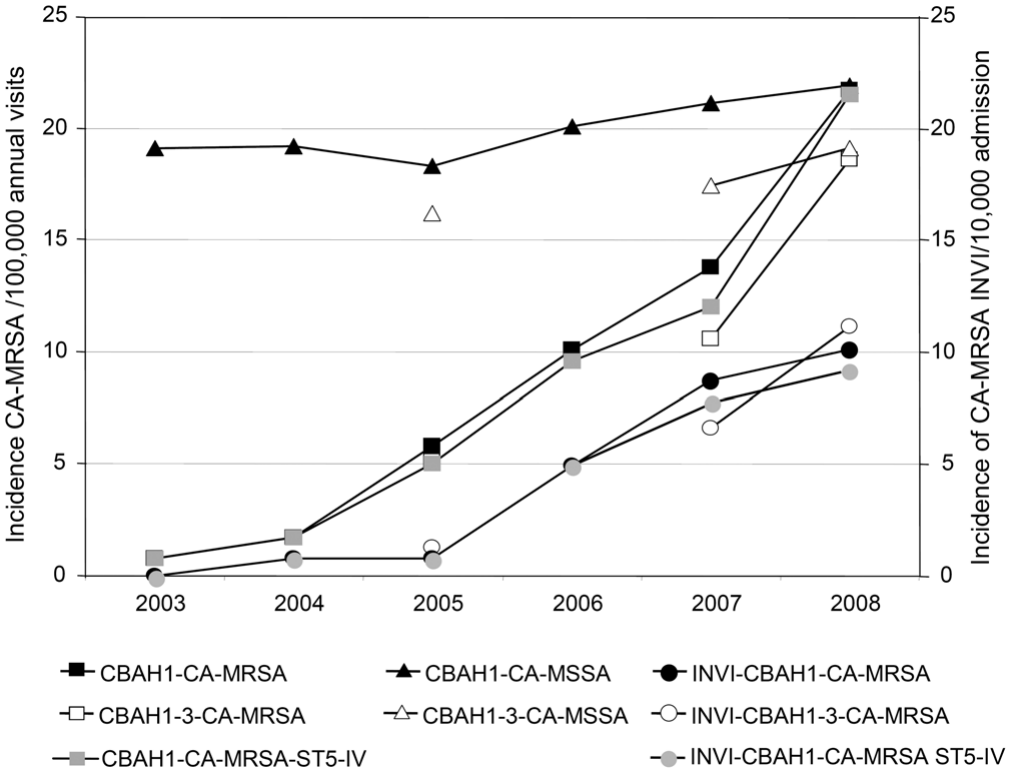
Evolution of community-associated *Staphylococcus aureus* infections from children in Cordoba-Argentina, 2003–2008. Evolution of the rates of all community-associated *Staphylococcus aureus* infections: *i)* methicillin-resistant *S. aureus* (CA-MRSA) infections [total (squares) and invasive-INVI (circles)], and *ii)* total methicillin-susceptible *S. aureus* (CA-MSSA) infections (triangles) in Córdoba children's hospitals, [H1 (CBAH1): 2003–2008: filled figures, and in H1, H2 and H3 (CBAH1-3): 2005 vs 2007–2008: empty figures] *iii)* methicillin-resistant community-associated *Staphylococcus aureus* infections caused by the ST5-IV-PVL^+^clone [total (gray squares) and invasive (INVI) (gray circles)] in H1 (CBAH1): 2003–2008.

During 2007–2008, CA-MRSA accounted for 45% (99 CA-MRSA/221 CA-*S. aureus*) of community-associated *S. aureus* infections in pediatric patients cared for in three children's hospitals of Córdoba, [38% (36/94) in 2007 and 49% (63/127) in 2008]. Hence, these data indicate a significant increase of the annual prevalence of CA-MRSA infections since 2005 [33% (7/21) for a three-month period [12] and 25% (18/71) annually, *P*<0.01 for trend]. Additionally, the overall rate of CA-MRSA infections increased significantly (3.6 fold) accounting for 5.1 to 18.6 cases/100,000 annual visits in 2005 and 2008, respectively, *P*<0.0001 (Figure 1). In contrast, the incidence of CA-MSSA infections only increased 1.1 fold, from 16.2 cases in 2005 to 19.1 cases/100,000 annual visits in 2008. The number of children with invasive CA-MRSA infections in three Córdoba children's hospitals increased significantly from 1.3 in 2005 to 11.1 cases/10,000 admissions in 2008, *P*<0.001.

Similar trends were observed yearly in one of these hospitals (CBAH1) upon analysis of the incidence (CA-MSSA, total CA-MRSA and invasive CA-MRSA infections, Figure 1) from 2003 to 2008.

The characteristics, origin and incidence of CA-MRSA during 2007 in all hospitals are shown in Table 1. Importantly, the rates of CA-MRSA infections were highly variable among children from central, eastern and northern regions of Argentina, reaching an overall rate of 14.2 cases/100,000 annual visits (range 6.9–27) in 2007 (Table 1).

**Table 1:** Characteristics, incidence of CA-MRSA infections and location of children's hospitals of northern, eastern and central of Argentina, 2007.

Children's Hospitals	Province	Number of beds	Annual admission	Annual visits^b^	Number of CA-MRSA^a^ 2007	Incidence^c^
H1	Córdoba	230	10,375	119,365	16	13.4
H2	Córdoba	75	3,083	123,000	10	8.1
H3	Córdoba	70	1,775	107,000	10	9.3
H4	Santa Fe	96	8,000	220,000	29	13.2
H5	Buenos Aires	274	6,756	202,000	14	6.9
H6	Buenos Aires city surrounding areas	142	7,539	264,938	33	12.5
H7	Santa Fe	178	13,169	175,683	48	27.3
H8	Autonomous city of Buenos Aires	485	18,427	296,452	53	17.9
H9	Autonomous city of Buenos Aires	228	8,917	252,083	42	16.7
H10	Corrientes	152	8,707	181,671	28	15.4
H11	Chaco	78	3,273	214,091	24	11.2

a Number of all CA-MRSA isolates detected in the three Cordoba children's hospitals during 2007 and those recovered in each hospital from the surveillance study for community onset *S. aureus* infections in children from Argentina-(CSACHARG) [16].

b annual visits: include outpatient facility and emergency service.

c Incidence: Number of cases/100,000 annual visits.

### Community onset MRSA infections: Demographic and clinical data

The distribution of CA-MRSA infections (Córdoba, 2007–2008 and CSACHARG, 2007) and HACO-MRSA infections (CSACHARG, 2007) regarding patient's clinical features, age and sex are shown in Table 2. Although children from Córdoba with CA-MRSA infections were significantly older than children from CSACHARG, both groups were comparable in sex, type and severity of the infections (Table 2).

**Table 2:** Demographic and clinical characteristics of children with Community-onset methicillin resistant *S. aureus* infections in central, northern and eastern regions of Argentina.

Characteristics	CA-MRSA CBAH1-H3^a^	CA-MRSA CSACHARG^b^	*P* c	CA-MRSA Total CBAH1-H3^a^ and CSACHARG^b^	HACO-MRSA CSACHARG^b^	*P* c
Period	2007–2008	2007			2007	
n(%), [n]	n: 99(%), [n]: 5	n: 213(%), [n]: 25		n: 312(%), [n]: 30	n: 51(%), [n]: 4	
Age, median of years (range)	6.1 (0.24–18)	3.4 (0.08–16)		4.1 (0.08–18)	3.2 (0.08–18)	
Sex (males)^d^	61(61)	128(60)	0.93	189(60)	30(59)	0.88
**Type and Severity of Infection** d						
***Skin and soft-tissue (SSTI)***						
*Abscess and cellulites* e	72(73)	136(64)	0.11	209(67)	6(12)	<0.001
*Necrotizing Fasciitis*	1(1)	-		1(0.5)	-	
**Surgical Site (SSI)**	-	-		-	12(23)	
***Musculoskeletal***	16(16), [2]	32(15), [11]	0.26	48(15), [13]	6(12), [2]	0.5
*Osteomyelitis*	11(11), [2]	17(8), [10]		28(9), [12]	5(10), [1]	
*Septic arthritis*	5(5)	13(6)		18(5.5)	-	
*Pyomyositis*	-	2(1), [1]		2(0.5), [1]	1(2), [1]	
***Respiratory***	5(5), [1]	23(11), [7]	0.09	28(9), [8]	9(17), [1]	0.06
*Pneumonia*	5(5), [1]	11(5), [2]		16(5), [3]	6(12), [1]	
*Pleural abscess*	-	12(6), [5]		12(4), [5]	3(6)	
***Bacteremia***	2(2)	15(7)	0.06	17(5.5)	12(23)	<0.001
***Endocarditis***	1(1), [1]	1(0.5), [5]		2(0.5), [6]	[1]	
***Pericarditis***	-	1(0.5)		1(0.5)	-	
***Deep abscess*** f	[1]	4 (1.5), [1]		4(1), [2]	2(4)	
***Lymphadenitis***	2(2)	1(0.5)		3(1)	1(2)	
***Meningitis***	-	[1]		[1]	3(6)	
**Invasive Infection** d	27(27)	77(36)	0.12	104(33)	33(65)	<0.001
**Sepsis** d	11(11)	29(13)	0.38	40(13)	14(27)	0.008

CA-MRSA: Community-associated methicillin resistant *Staphylococcus aureus*, HACO-MRSA: Healthcare-associated community-onset methicillin resistant *Staphylococcus aureus* infections.

a CBAH1-H3: Prospective surveillance of CO-*S. aureus* infections in children from three children's hospitals of Córdoba (CBAH1, CBAH2 and CBAH3), 2007 and 2008.

b CSACHARG: Prospective surveillance of CO-*S. aureus* infections in children from Argentina, 2007 [16].

c *P* values are based on chi-square test or Fisher's exact test, as appropriate, for CA-MRSA,CBAH1-H3 vs. CA-MRSA, CSACHARG and total CA-MRSA vs. HACO-MRSA comparisons by each of categorical variables (males and infection type); *p*<0.05 was considered statistically significant.

d Values are number of patients and the percentages are indicated in parentheses. [n]: number of patients with this secondary infection focus (CA-MRSA: 342 infections in 312 patients).

e Abscess and cellulites: include 9 cases of impetigo(1 from CBAH1-H3 and 8 cases from CSACHARG).

f Deep abscess included: breast (2 cases), psoas (4 cases), liver and renal (1 case each one) abscesses.

Similar types of invasive CA-MRSA infections (n: 7) were detected in CBAH1 during 2003–2006: osteomyelitis (n: 2), arthritis (n: 1), pneumonia (n: 1), bacteremia (n: 1), lymphadenitis (n: 1) and necrotizing fasciitis (n: 1).

Among selected children with HACO-MRSA infections (n: 51), multiple risk factors (≥2) were found in 35 (68%). The most common HRFs were: presence of indwelling catheters or percutaneous medical devices (85%) and hospitalization within the previous year (73%). In addition, 70% of the patients had chronic diseases, including immunodeficiencies (human immunodeficiency virus-HIV/acquired immunodeficiency syndrome-AIDS and malignancies), cardiovascular diseases, chronic pulmonary diseases, chronic renal insufficiency, diabetes, or chronic skin illnesses. These children were more likely to develop invasive infections (65% vs 33%) and presentation with sepsis (27% vs 13%) than children with CA-MRSA infections. Invasive HACO-MRSA cases included mainly lungs and bloodstream infections. In addition, they had significantly lower frequency of SSTI infections (12% vs 67%).

Moreover, 23% of HACO-MRSA isolates were obtained from surgical site infections (Table 2).

The isolates from invasive CA-MRSA infections from CBAH1-H3 and CSACHARG groups (total n: 104) were obtained from blood (74%), other normally sterile body sites including joints (8%), bone (8%), pleural fluid (6%), other body fluids (2%) and other sites (1% each).

### Antimicrobial susceptibility profile of community-onset MRSA strains

From all CA-MRSA isolates (n: 312) recovered from the CBAH1-3 and CSACHARG groups during 2007–2008, 20% were resistant to at least one non-β-lactam antibiotic. Resistance to erythromycin (ERY-15%), clindamycin (CLI-14%, 93% of inducible resistance); gentamicin (GEN-8%), rifampin (RIF-2%) and chloramphenicol (CHL-1%) were detected. The HACO-MRSA isolates showed significantly (*P*>0.001) higher rates of resistance to ERY (45%), CLIN (42%, 23% inducible); GEN (43%) and RIF (14%) than the CA-MRSA isolates. In addition, resistance to ciprofloxacin (22%) and to trimethoprim-sulfamethoxazole (6%) were only detected among HACO-MRSA isolates. All isolates were susceptible to vancomycin by agar dilution method (MIC90, 1 µg/mL; MIC range, 0.25–2 µg/mL) and teicoplanin (MIC90, 2 µg/mL; MIC range, 0.25–4 µg/mL). From the total 363 CO-MRSA isolates evaluated, only 3 MRSA from children with HRFs (HACO-MRSA infections), were found to be h-VISA.

### Molecular characterization of community-onset MRSA strains: CA-MRSA and HACO-MRSA

The molecular characteristics [sequence type (ST, as defined by MLST), clonal complex (CC), *agr* allotype, PFGE, SCC*mec* and *spaA* types, presence of *pvl* genes and virulence genes profile, along with the drug resistance pattern of the most predominant pulsotypes are shown in Table 3. The distribution of the most predominant genotypes of CO-MRSA isolates involved in invasive and non invasive infections according to the epidemiologic case classification (CA-MRSA and HACO-MRSA) are shown in Table 4. The molecular characterization of the three h-VISA strains demonstrated that they belonged to three different MRSA clones (Table 3).

**Table 3:** Characteristics of MRSA clones isolated from children with community onset MRSA infections (CO-MRSA), Argentina.

	Related to Pediatric clone			Southwest Pacific (SWP) clone	South American USA300 clone	Cordobes/Chilean clone	Brazilian clone
Clone	CA-MRSA clone		HA-MRSA clone				
	n: 285		n: 10	n: 39	n: 4	n: 12	n: 3
Molecular characteristics							
***agr*** allelic group	2		2	3	1	2	1
**ST** n (%)^a^	ST5: 283 (99); ST1524:2 (1)		ST100: 10 (100)	ST30: 35 (90); ST196: 4 (10)	ST8: 4 (100)	ST5: 12 (100)	ST239: 3 (100)
**Clonal complex**	5		5	30	8	5	8
**SCC** ***mec*** type n (%)^a^	IV(2B): IVa: 275 (96), IVc: 6 (2), IVNT^e^: 1; Vr^f^: 2 (0.7); NT: 1 (0,3)		IVNv: 10 (100)	IV(2B): IVc: 38 (97), IVa: 1 (3)	IV(2B): IVc: 3 (75), IVa: 1 (25)	I	IIIA
**PFGE type**	I		C	N	USA300	A	B
***pvl***: n (%)^b^	PVL^+^ 259 (91)	PVL^−^ 26 (9)	PVL^−^10 (100)	PVL^+^ 39 (100)	PVL^+^ 4 (100)	PVL^−^12 (100)	PVL^−^3 (100)
**PFGE Subtype**: n (%)^a^	I-1: 202 (71) and 14 minor subtypes	I-2: 7 (2.5) and 12 minor subtypes	C1: 2 (20) and 7 minor subtype	N4: 26 (67) and 3 minor subtypes	USA300, 4 subtypes	A1: 4 (33) and 8 minor subtypes	B15 2 (66) and 1 subtype
**RIDOM ** ***spa*** type: n (%)^a^	t311: 259 (100)	t002: 22 (85); t311, t045; t067; t1094	t002: 10 (100)	t019: 29 (72); t021: 5 (13); t6653: 4 (10); t975	t008: 4 (100)	t149: 12 (100)	t037: 3 (100)
**Virulence genes profile** c	*sea-egc-lukDE*	*egc-lukDE*	*egc-lukDE*	*egc-lukDE-bbp-cna*	*lukDE-sek-bsa*: 3 (75); *lukDE-sek-sed-sej- bsa*: 1 (25)	*egc-lukDE*	*lukDE-bsa*
**Drug resistance non-β-Lactam** (%)^d^	ERY(14)CLINi (14)GEN (8)RIF (1)CHL (1)**h-VISA** (1)		GEN (100)ERY (50)CLINi (10); CLINc (30)RIF (30)**h-VISA** (1)	GEN(5)ERY(18)CLINi (18)CHL(3)	GEN(25)	GEN (92)CIP (75)ERY(92)CLIc (92)RIF (17)	GEN (100)CIP (100)ERY (100)CLI (100)RIF(6)CHL (100)SXT (100)**h-VISA** (1)
**MINOR CLONES**, n: 10							
ST188/CC1, n: 2	PFGE type T1, SCC*mec*Vr, *spa*-t189, *agr* 1, *cna* ^+^, PVL**^+^**						
ST917/CC8, n: 4	PFGE type R1, SCC*mec*IVNT, *spa*-t148, *agr* 1, PVL^−^						
ST207/CC509, n:3	PFGE type Y1, SCC*mec*IVa, *spa*-t525, *agr* 3, PVL^−^						
ST918/Singleton, n:1	PFGE type S1, SCC*mec*IVa, *spa*-t1194, *agr* 3, PVL^−^						

*agr* type, type of accessory gene regulator, ST: Sequence Type, SCC*mec*: Staphylococcal Cassette Chromosome *mec*, PFGE, Pulsed Field Gel Electrophoresis; RIDOM *spa* type: staphylococcal protein A (*spa*) type assigned through the RIDOM databases (http://spaserver.ridom.de).

a n (%), total number and % of strains with this molecular characteristic [PFGE subtype (only those more frequent are indicated) or ST or *spa* type or *SCCmec* type]. % is not expressed when only one isolate with this characteristic was detected.

b *pvl*, Panton-Valentine leukocidin genes (*lukS*-PV-*lukF*-PV); indicated as number and % of isolates harboring (PVl^+^) or not (PVL^−^) *pvl* genes.

c virulence genes profile: From all virulence genes analyzed, only those detected are indicated (number and % of positive isolates is expressed when not all isolates harbor this virulence factor).

d Drug resistance to non-β-Lactams (%), is indicated as follows: Gentamicin (GEN), Ciprofloxacin (CIP), Erythromycin (ERY), Clindamycin (CLIc and CLIi: constitutive and inducible resistance to macrolides, lincosamide and streptogramine B, respectively), rifampin (RIF), chloramphenicol (CHL), trimethoprim/sulfamethoxazole (SXT) and minocycline (MIN) (%) of strains resistant to these antibiotics within each pulsotype is indicated when more than one isolate was detected. h-VISA (1): means one isolate belonging to this clone with phenotype h-VISA.

e IV NT: SCC*mec* type IV non typable.

f Vr: SCC*mec* related to V.

**Table 4:** Genotypes of methicillin resistant *S. aureus* (MRSA) isolates recovered from children with Community-onset infections (invasive and non-invasive) in central, northen and eastern regions of Argentina, by epidemiologic case classification.

	Community onset (CO), N° (%) of cases & N° (%) of INVI isolates in indicated epidemiologic class					
	Total CO	Community–Associated (CA)				Healthcare-associated CO (HACO)
**Genotypes** c		CBAH1-H3^a^ 2008	CBAH1-H3^a^ 2007	CSACHARG^b^ 2007	Total CA	CSACHARG^b^ 2007
**I-ST5-IV**	285 (79); 99 (72)	57 (90); 14 (82)	29 (80); 7 (70)	170 (80);61 (79)	256 (82);82 (79)	29 (57);17 (51)
**N-ST30-IV**	39 (11), 11 (8)	3 (5), 2 (12)	3 (8); 1 (10)	30 (14); 7 (9)	36 (11.5); 10(10)	3 (6); 1 (3)
**USA300-ST8-IV**	4 (1); 3 (2)	2 (3); 1 (6)	1 (3);1 (10)	1 (0.5), 1 (1)	4 (1), 3 (3)	—
**C-ST100-IVNv**	10 (3); 6 (4)	—	1 (3); —	4 (2); 2 (2.5)	5 (2); 2 (2)	5 (10); 4 (12)
**A-ST5-I**	12 (3); 10 (7)	—	1 (3); 1 (10)	—	1 (0.5); 1 (1)	11 (21); 9 (27)
**B-ST239-IIIA**	3 (1); 2 (1.5)	—	—	—	—	3 (6); 2 (6)
**OTHERS**	10 (2)	1 (2)	1 (3)	8 (3.5)	10 (3)	—
**Total**	n: 363 & INVI: 137	n: 63 & INVI: 17	n: 36 & INVI: 10	n: 213 & INVI: 77	n: 312 & INVI: 104	n: 51 & INVI: 33

**INVI**: Invasive infections.

a CBAH1-H3: Prospective surveillance of CO-*S. aureus* infections in children from three children's hospitals of Córdoba (CBAH1, CBAH2 and CBAH3) 2007 and 2008.

b CSACHARG and: Prospective surveillance of CO-*S. aureus* infections in children from Argentina, 2007 [16].

c Genotypes are denoted as: type (by PFGE)-Sequence Type (ST by MLST)-SCC*mec* type.

#### CA-MRSA clone ST5-IV

PFGE performed on 363 isolates (312 CA-MRSA and 51 HACO-MRSA) revealed a predominant major pulsotype, (“I”; 79%, 285/363 isolates) with a more frequent subtype, I-1 (71%, 202/285). All isolates belonging to the pulsotype “I” were characterized as *agr* 2, ST5, except two isolates belonging to a new single locus variant (SLV) of ST5: ST1524, CC5 (Clonal Complex), *spa*-t311 (91% of isolates) and related (t002, t045; t067; t1094). Most of them were associated with SCC*mec* type IV(2B), 96% with subtype IVa:, 2% with subtype IVc and one isolate was nominated as SCC*mec* type IV non typable (IVNT), since it was positive by PCR for *ccrB2* and *mec* gene complex class B, but negative for J1 regions: a, b, c, d, g and h (Table 3). Two isolates (I18 subtype) were positive only for *ccrC* locus and negative for J1 region of SCC*mec* V and for other SCC*mec* regions analyzed [31] so they were tentatively designated as SCC*mec* related to V (Vr) (Table 3).

**Figure 2:**
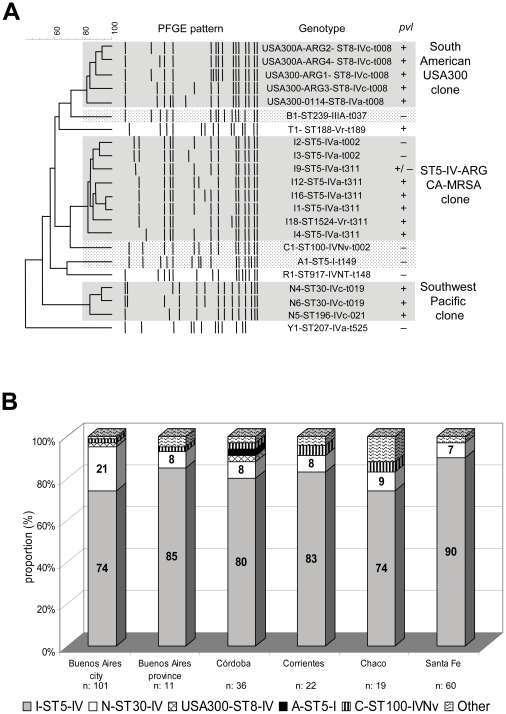
Molecular characteristics and proportion from different regions of Argentina of dominant community methicillin-resistant-*Staphylococcus aureus* clones. **A**. PFGE pattern analysis for representative isolates belonging to the most prevalent subtypes of community-onset-MRSA clones (CA-MRSA and HA-MRSA) detected in central, eastern and northern regions of Argentina during 2007–2008. The schematic presentation of *SmaI* restriction patterns (middle) and dendrogram (left) by the unweighted-pair group method using average linkage clusterings are shown. Genotypes are denoted as subtype (by PFGE)-ST (by MLST)-SCC*mec* type-*spa* type (right). CA-MRSA clones appear in gray. The presence (+) or absence (−) of *pvl* genes (by PCR) is also indicated for each subtype; strains with and other without *pvl* genes belonging to the same PFGE subtype (I9) are indicated as +/−. The PFGE pattern of USA300-0114 (ST8-IVa-*t008*-ACME+) is shown for comparison purposes. The first (A1-ST5-I-t149-Cordobes/Chilean), second (B1-ST239-IIIA-t037-Brazilian) and third (C1-ST100-IVNv-t002-Pediatric) more frequent HA-MRSA clones in our country, detected among community-onset MRSA infections, are also shown (dotted gray). **B**: Proportion of CA-MRSA clones among representative isolates from different regions of Argentina in 2007.

The *pvl* genes were not detected in 9% of the “I” pulsotype isolates (26/285) (Table 3 and Figure 2A). There was considerable diversity among the PFGE patterns of these isolates, demonstrating an ongoing evolution: 13 subtypes from 26 PVL^−^ isolates vs 15 subtypes from 259 PVL^+^ isolates. Unlike PVL^+^ isolates, within the PVL^−^ group, *spa*-t002 (85%) was much more prevalent than *spa*-t311 (4%); and did not have the *sea* gene.

Additionally, all isolates belonging to pulsotype “I” recovered in CBAH1 during 2003–2006 (n: 20) were characterized as ST5, *spa*-t311, associated with SCC*mec*IVa as well as they harbour *pvl* and *sea* genes.

This clone represented 79% of all community-onset MRSA isolates (285/363) in both groups of infections, CA-MRSA (82%, 256/312) and HACO-MRSA (57%, 29/51) (Figure 2 and Table 4). Moreover, this pulsotype predominated among CA-MRSA isolated in CBAH1 during 2003–2006 (91%, 20/22). Longitudinal analysis of CA-MRSA infections at this hospital demonstrated that the significant increase of the incidence of both total CA-MRSA infections and invasive CA-MRSA infections were due to the dissemination of this single pulsotype “I” (ST5-SCC*mec*IV-PVL^+^) increasing from 1 to 25 cases of CA-MRSA infections between 2003 and 2008 (Figure 1).

On the other hand, both groups, PVL^+^ and PVL^−^ variants, produced invasive infections in similar proportions (35%, 90 invasive infections/259 PVL^+^ MRSA infections vs 35%, 9 invasive infections of 26 pulsotype “I”-PVL^−^ MRSA infections; *P = 0.*96). Moreover, both groups were comparable regarding sepsis presentation (13%, 33/259 PVL^+^ vs 12%, 3/26 PVL^−^; *P = 0*.8).

#### Southwest Pacific (SWP) clone

The second prevalent clone among total community-onset MRSA isolates (11%, 39/363) was the N pulsotype or Southwest Pacific (SWP) clone. It was characterized as *agr* 3, ST30 (4 isolates as ST196/SLV) belonging to CC30, *spa*-t019 and related t021, t6653 (new *spa* type described for the first time in this study) and *spa*-t975. Most isolates harbored SCC*mec*IVc, (97%) and all of them carried the *pvl* genes. This genetic background accounted for 11.5% (36/312) and 6% (3/51) of CA-MRSA and HACO-MRSA isolates respectively (Table 4). The molecular characteristics of this clone and its distribution in different regions of Argentina during 2007 are shown in Table 4 and Figure 2. In addition, this clone was significantly more frequent in Buenos Aires city and surrounding areas (21%) than in other regions of Argentina (average 8%, range = 7−9, *P* = 0.009, Figure 2B).

#### Pediatric clone in Argentina: ST100-IVNv

A minor HA-MRSA clone, non-multiresistant to antibiotics, named pulsotype C or D by Sola et al and Corso et al, respectively, clonally related to the Pediatric clone by PFGE analysis [36], [37] was also detected. It was characterized as *agr* 2, ST100, belonging to CC5, *spa*-t002, associated with a SCC*mec*IV new variant (SCCmec IVNv), and PVL^−^ [12], [15].

In this study, *Tn*4001 was detected, integrated into the class B *mec* complex upstream of the *mecA* gene (class B2 *mec* gene complex) by means of PCR typing of different regions of SCC*mec*IV in representative isolates of the most frequent subtypes (File S1). Hence, this new variant of SCC*mec*IV, in addition to class B2 *mec* gene complex, also carries type 2 *ccr* gene complex and a J1 region with homology to SCC*mecIVc*, but unlike the SCC*mec*IV(2B&5), it lacks of an SCC with *ccrC* and contains *dcs* gene.

This clone was significantly more frequent among HACO-MRSA infections (10%, 5/51) than among total CA-MRSA infections (2%, 5/312), *P* = 0.009, (Table 4).

#### South American USA300 clone

Four CA-MRSA isolates (one detected in Buenos Aires in 2007 and three in Córdoba during 2007–2008) were clonally related by PFGE to the USA300 MRSA epidemic strain. They belonged to ST8, CC8, *spa*-t008 and to *agr* 1. Three of these isolates were associated with SCC*m*ecIVc and one, with subtype IVa. All of them carried the *pvl*, *bsaA* and *seK* genes. In contrast, all lacked of the *arcA* gene, which is an indicator of the presence of the ACME. The isolate associated with *SCCmec*IVa also carried *sed* and *sej* genes (Figure 2A and Table 3).

#### Other minor clones

Among CA-MRSA isolates, other minor pulsotypes were also detected accounting cumulatively for only 4% of isolates (n: 10, less than 2% each), (Table 3). Among these, two isolates were characterized as ST188/CC1 and associated with SCC*mec*Vr constituting the first time that this genotype is reported in America [19].

The MRSA strains traditionally associated with nosocomial infections in Argentina [12], [15], [36], [37] only accounted for 37% of the total HACO-MRSA infections (Cordobes/Chilean-pulsotype A-ST5-I-t149, 21%; Pediatric-pulsotype C-ST100-IVNv-t002: 10% and Brazilian-pulsotype B-ST239-IIIA-t037: 6%, Figure 2A and Table 4).

## Discussion

Laboratory-based surveillance of community-associated *S. aureus* infections in three children's hospitals in Córdoba-Argentina during 2007–2008 revealed a significant increase of the annual prevalence of CA-MRSA infections since 2005 (49%, 2008 vs 25%, 2005). Moreover, the incidence of CA-MRSA infections among children tripled over that time frame, associated with an 8.5-fold increase in invasive CA-MRSA infections, despite the almost stable overall rate of CA-MSSA infections (1.1 fold increase). A similar pattern with a steady annual increase of CA-MRSA infections and a slight increase of CA-MSSA infections was detected in one (H1) of these hospitals between 2003 and 2008. Hence, we demonstrated that in Córdoba, Argentina, as reported in some regions of United States, particularly in patients with skin and soft-tissue infections, CA-MRSA strains are adding to the burden of CO-*S. aureus* infections, instead of replacing other *S. aureus* strains [8], [38].

Unlike in North America, few studies have documented the epidemiology of MRSA infections in the community among Latin-American children [1], [12], [39]. This study provides the first comprehensive description carried out in Argentina about the evolution of CA-MRSA infections over time (2003–2008) in children from Córdoba.

The types of CA-MRSA infections detected in this study are similar to those described worldwide. Nevertheless, it is important to remark the high proportion of children with invasive CA-MRSA infections in Argentina (33%) compared to previous reports from other countries (1–12%) [20], [40]. Regarding HACO-MRSA infections, most children had some type of chronic disease and multiple HRFs, were more susceptible to surgical site and invasive infections as well as sepsis, and had significantly lower frequency of SSTIs compared to children with CA-MRSA infections, in agreement with other studies [4], [21], [41].

Although variable rates of CA-MRSA infection among children from different regions of Argentina were detected in 2007, one of the most striking finding of our study is that one major CA-MRSA clone (I-ST5-SSC*mec*IV-PVL^+^) is currently predominanting in both groups: community-associated cases (82%) and healthcare-associated, community-onset cases (57%) in hospitals of northern, eastern and central regions of our country, contrasting with other countries [4].

Therefore, the wide dissemination of this clone over all these regions of Argentina, along with the continued steep rise of its incidence over time in Córdoba, confirm its epidemic nature. Importantly, these results suggest that many children, despite documented contacts with healthcare delivery, are frequently infected with a CA-MRSA clone. This finding, though indirectly, suggests that this clone has entered into the healthcare settings and thus, represents a potentially serious public health challenge for next years, as described elsewhere for other CA-MRSA clones [8]–[10].

The molecular characteristics shared by the isolates belonging to this major clone corresponded largely to those reported in our previous paper [12], namely, *agr*2, ST5, *spa*-t311 and related ones, *SCCmec*IV(2B) (96% IVa). In addition, most of these isolates carried *pvl* and *sea* genes (91%). Although most of CA-MRSA infections (invasive and non invasive) caused by the “I” clone have been produced by PVL^+^ isolates, both groups, PVL^+^ and PVL^−^, were comparable regarding the proportions of invasive infections and presentation with sepsis. Hence, these results lead to hypothesize that the presence of *pvl* and *sea* genes associated with *spa*-t311 in this lineage would be more related to a possible increase in transmissibility and fitness of this CA-MRSA clone, rather than to its virulence. Further studies are needed to confirm this hypothesis.

Recently, Nübel et al [42], by using single-nucleotide polymorphism analysis, demonstrated that much of the population structure of ST5 is local, with multiple independent introductions of SCC*mec*, producing a regional-specific evolution of particular sublineages. In agreement with that hypothesis, in previous studies we demonstrated the presence of ST5-PVL^+^ lineages among MSSA strains in our country [12], [43]. In this study, 9% of the isolates belonging to I pulsotype were PVL^−^. Additionally, among the isolates characterized as I9 subtype-*spa*-t311-SCC*mec*IVa, we also found one PVL^−^ variant and another harboring SCC*mec*IVc (Figure 2A). Together, these results support the hypothesis about multiple independent acquisitions of different SCC*mec* types (IVa or IVc or Vr) and virulence genes (*pvl* and *sea*) by a common ST5 MSSA ancestor.

On the other hand, some studies have described the ST5-SCC*mec*IV-PVL^+^ genotype among CA-MRSA isolates in United States and some countries of Europe and Africa. However in all of them, the isolates had different profiles of virulence genes and/or resistance to antibiotics and so far, its role as dominant genetic background of CA-MRSA over time has not been duplicated outside Argentina [3], [44]–[49].

Accordingly, although this clone is genetically related to the international HA-MRSA “classical” Pediatric clone (ST5-SCC*mec*IV) identified in several countries [50], based on the molecular characterization (CA-MRSA and CA-MSSA) and clinical epidemiology over time, we suggest that this clone is a new epidemic CA-MRSA, probably endowed with increased fitness in this country. Hence, the possibility of being an escaped HA strain should be ruled out.

Conversely, one of the three most predominant HA-MRSA clones in Argentina since 1998 was clonally related to the Pediatric clone by PFGE analysis [36], [37] which was characterized as ST100-SCC*mecIV* related to IVc-*spa*-t002-PVL^−^ [15]. In Córdoba, some community-onset infections were caused by this HA-MRSA clone before the emergence of the new CA-MRSA clone, probably as an “escaped” HA-MRSA strain [12], [15]. Accordingly, it was more commonly isolated from HACO-MRSA cases than from CA-MRSA infections (10% vs 2%), as shown herein. The ST100 genotype was also identified in a MRSA isolate from Zurich in 2003 (MRSA-ZH47), associated with the new SCC*mec*IV(2B&5) [35]. In this study, we demonstrated for the first time that the SCC*mec* associated with pulsotype C (Pediatric clone)-ST100 in Argentina is closely related to SCC*mec*IV(2B&5). Therefore, to classify this new variant of SCC*mec*IV, it would be necessary to determine the complete nucleotide sequence, which is currently in progress.

The pandemic Southwest Pacific-(SWP) clone or USA1100 was one of the minor clones found, accounting for 11.5% of CA-MRSA isolates. It was characterized as pulsotype N, ST30, *spa*-t019 and related ones. Most isolates harbored SCC*mec*IVc (97%) all of them carried *pvl* genes and adhesins *cna* and *bbp* genes. Since mid-1990s, this CA-MRSA clone, ST30-IV-PVL^+^ with different pulsotypes or *spa* types or SCC*mec*IV subtypes has been reported from many regions of the world [1], [46]. In Buenos Aires, this clone (ST30-IV) had already been detected as a minor one among CA-MRSA isolates in 2004–2006 [13]. In our study, this clone was significantly more frequent in Buenos Aires city and surrounding areas (21%) than in other regions analyzed, where it accounted for 8% of CA-MRSA isolates in 2007. Although its frequency is still very low, this situation might suggest that this CA-MRSA clone is spreading from Uruguay, where it is highly prevalent at community and hospital settings [11], [51].

Recently, the South American USA300 MRSA-(ST8-*SSCmec*IV-*spa*t008-PVL^+^-ACME^−^) clone was described as the most predominant (96%) CA-MRSA in northern areas of South America between 2006 and 2008 [14]. An additional important issue of this study was the finding of the first four cases of CA-MRSA infections caused by this highly virulent CA-MRSA clone in the southern cone of Latin America. ACME-negative variants of USA300 also appear to be common in Western Australia and in Spain [52]–[54], though we were unable to find eventual epidemiological association between Argentinean strains and those from these countries.

Our results confirmed the high prevalence of MRSA infections in the community in Argentinean children, so the characteristics of antimicrobial susceptibility of these strains must be considered for the selection of empiric therapies. Although most CA-MRSA isolates were susceptible to all non-β-lactam antibiotics, inducible resistance to clindamycin (14%), resistance to gentamicin (8%), rifampin (1%) and chloramphenicol (1%) were detected in isolates belonging to ST5-IV-PVL^+^ CA-MRSA clone (Table 3). It is important to remark that this clone's resistance to antibiotics was only represented by clindamycin, just in 10% of isolates, as described in the previous surveillance performed in Córdoba in2005 [12]. Hence, these results support the ability of the ST5-IV-PVL^+^ CA-MRSA clone to acquire new resistance determinants. On the other hand, the higher rates of resistance to non-β-lactam antibiotics detected among HACO-MRSA strains were associated with traditional HA-MRSA clones in our country: the Cordobes/Chilean, Brazilian and Pediatric clones. In addition, according to these results, it would be possible to infer that the presence of HRFs should be considered for selection of empirical therapy in children with community-onset MRSA infections.

Importantly, in our study, three strains with h-VISA phenotype from children with HRFs were detected. One of these cases and another one detected in an adult patient without HRFs in 2009 [55] were PVL^−^ variants of ST5-SCC*mec*-IV CA-MRSA clone. Considering the high proportion of children with invasive CA-MRSA infections caused by this clone, along with the fact that vancomycin is still the centerpiece used for the treatment of this type of CA-MRSA infections [7], this finding constitutes another concern for public health.

Our study was limited by the use of the CDC criteria to identify patients with infections caused by CA-MRSA isolates, which appear to underestimate the burden of diseases produced by these strains. Additionally, evidence from this study, though indirect, indicate that CA-MRSA strains have entered the hospital settings of Argentina and probably the burden of CA-MRSA is even greater than shown here.

In conclusion, we observed a significant increase in the incidence of invasive and non-invasive CA-MRSA infections, in children of Córdoba between 2005 and 2008. This raise could be attributed entirely to the rapid dissemination of an epidemic CA-MRSA clone characterized as I-ST5-*SCCmec*IVa-*spa*-t311-PVL^+^. Importantly, this highly virulent clone with capacity to express the phenotype h-VISA, predominates in central, northern and eastern regions of Argentina and still more worryingly, it has probably entered the hospital settings. The specific virulence, antibiotic resistance and transmission traits of a CA-MRSA clone distinct from USA300 would need to be identified in order to recognize priority targets for drug and vaccine development. These virulent clones represent a new threat to public health in Argentina and probably worldwide, warranting close monitoring during next years.

## Supporting Information

File S1 Molecular characterization of SCC*mec* associated with PFGE pattern C and sequence type ST100: Pediatric clone in Argentina.
(PDF)

## References

[1] David MZ, Daum RS (2010). Community-associated methicillin-resistant *Staphylococcus aureus*: epidemiology and clinical consequences of an emerging epidemic.. Clin Microbiol Rev.

[2] Deurenberg RH, Stobberingh EE (2009). The molecular evolution of hospital- and community-associated methicillin-resistant *Staphylococcus aureus*.. Curr Mol Med.

[3] Deleo FR, Otto M, Kreiswirth BN, Chambers HF (2010). Community-associated meticillin-resistant *Staphylococcus aureus*.. Lancet.

[4] Limbago B, Fosheim GE, Schoonover V, Crane CE, Nadle J (2009). Characterization of methicillin-resistant *Staphylococcus aureus* isolates collected in 2005 and 2006 from patients with invasive disease: a population-based analysis.. J Clin Microbiol.

[5] Gonzalez BE, Martinez-Aguilar G, Hulten KG, Hammerman WA, Coss-Bu J (2005). Severe Staphylococcal sepsis in adolescents in the era of community-acquired methicillin-resistant *Staphylococcus aureus*.. Pediatrics.

[6] Howden BP, Davies JK, Johnson PD, Stinear TP, Grayson ML (2010). Reduced vancomycin susceptibility in *Staphylococcus aureus*, including vancomycin-intermediate and heterogeneous vancomycin-intermediate strains: resistance mechanisms, laboratory detection, and clinical implications.. Clin Microbiol Rev.

[7] Liu C, Bayer A, Cosgrove SE, Daum RS, Fridkin SK (2011). Clinical practice guidelines by the Infectious Diseases Society of America for the treatment of methicillin-resistant *Staphylococcus aureus* infections in adults and children.. Clin Infect Dis.

[8] Gerber JS, Coffin SE, Smathers SA, Zaoutis TE (2009). Trends in the incidence of methicillin-resistant *Staphylococcus aureus* infection in children's hospitals in the United States.. Clin Infect Dis.

[9] David MZ, Glikman D, Crawford SE, Peng J, King KJ (2008). What is community-associated methicillin-resistant *Staphylococcus aureus*?. J Infect Dis.

[10] Maree CL, Daum RS, Boyle-Vavra S, Matayoshi K, Miller LG (2007). Community-associated methicillin-resistant *Staphylococcus aureus* isolates causing healthcare-associated infections.. Emerg Infect Dis.

[11] Ma XX, Galiana A, Pedreira W, Mowszowicz M, Christophersen I (2005). Community-acquired methicillin-resistant *Staphylococcus aureus*, Uruguay.. Emerg Infect Dis.

[12] Sola C, Saka HA, Vindel A, Bocco JL, Cordoba MRSA Collaborative Study Group (2008). Emergence and dissemination of a community-associated methicillin-resistant Panton-Valentine leukocidin-positive *Staphylococcus aureus* clone sharing the sequence type 5 lineage with the most prevalent nosocomial clone in the same region of Argentina.. J Clin Microbiol.

[13] Gardella N, von Specht M, Cuirolo A, Rosato A, Gutkind G (2008). Community-associated methicillin-resistant *Staphylococcus aureus*, eastern Argentina.. Diagn Microbiol Infect Dis.

[14] Reyes J, Rincon S, Diaz L, Panesso D, Contreras GA (2009). Dissemination of methicillin-resistant *Staphylococcus aureus* USA300 sequence type 8 lineage in Latin America.. Clin Infect Dis.

[15] Sola C, Cortes P, Saka HA, Vindel A, Bocco JL (2006). Evolution and molecular characterization of methicillin-resistant *Staphylococcus aureus* epidemic and sporadic clones in Cordoba, Argentina.. J Clin Microbiol.

[16] Paganini H, Della Latta MP, Muller Opet B, Ezcurra G, Uranga M (2008). [Community-acquired methicillin-resistant *Staphylococcus aureus* infections in children: multicenter trial].. Arch Argent Pediatr.

[17] Rodriguez-Noriega E, Seas C, Guzman-Blanco M, Mejia C, Alvarez C (2010). Evolution of methicillin-resistant *Staphylococcus aureus* clones in Latin America.. Int J Infect Dis.

[18] Chua K, Laurent F, Coombs G, Grayson ML, Howden BP (2011). Antimicrobial resistance: Not community-associated methicillin-resistant *Staphylococcus aureus* (CA-MRSA)! A clinician's guide to community MRSA - its evolving antimicrobial resistance and implications for therapy.. Clin Infect Dis.

[19] Monecke S, Coombs G, Shore AC, Coleman DC, Akpaka P (2011). A Field Guide to Pandemic, Epidemic and Sporadic Clones of Methicillin-Resistant *Staphylococcus aureus*.. PLoS One.

[20] Kaplan SL, Hulten KG, Gonzalez BE, Hammerman WA, Lamberth L (2005). Three-year surveillance of community-acquired *Staphylococcus aureus* infections in children.. Clin Infect Dis.

[21] Klevens RM, Morrison MA, Nadle J, Petit S, Gershman K (2007). Invasive methicillin-resistant *Staphylococcus aureus* infections in the United States.. JAMA.

[22] Morrison MA, Hageman JC, Klevens RM (2006). Case definition for community-associated methicillin-resistant *Staphylococcus aureus*.. J Hosp Infect.

[23] Klevens RM, Morrison MA, Fridkin SK, Reingold A, Petit S (2006). Community-associated methicillin-resistant *Staphylococcus aureus* and healthcare risk factors.. Emerg Infect Dis.

[24] Goldstein B, Giroir B, Randolph A (2005). International pediatric sepsis consensus conference: definitions for sepsis and organ dysfunction in pediatrics.. Pediatr Crit Care Med.

[25] Clinical and Laboratory Standards Institute..

[26] Wootton M, MacGowan AP, Walsh TR, Howe AR (2007). A multicenter study evaluating the current strategies for isolating *Staphylococcus aureus* strains with reduced susceptibility to glycopeptides.. J Clin Microbiol.

[27] Hiramatsu K, Hanaki H, Ino T, Yabuta K, Oguri T (1997). Methicillin-resistant *Staphylococcus aureus* clinical strain with reduced vancomycin susceptibility.. J Antimicrob Chemother.

[28] Satola SW, Farley MM, Anderson KF, Patel JB (2010). Comparison of Detection Methods for Heteroresistant Vancomycin Intermediate *Staphylococcus aureus* (hVISA) using Population Analysis Profile as the Reference Method.. J Clin Microbiol.

[29] Enright MC, Day NP, Davies CE, Peacock SJ, Spratt BG (2000). Multilocus sequence typing for characterization of methicillin-resistant and methicillin-susceptible clones of *Staphylococcus aureus*.. J Clin Microbiol.

[30] Harmsen D, Claus H, Witte W, Rothganger J, Claus H (2003). Typing of methicillin-resistant *Staphylococcus aureus* in a university hospital setting by using novel software for spa repeat determination and database management.. J Clin Microbiol.

[31] Milheirico C, Oliveira DC, de Lencastre H (2007). Update to the multiplex PCR strategy for assignment of mec element types in *Staphylococcus aureus*.. Antimicrob Agents Chemother.

[32] Okuma K, Iwakawa K, Turnidge JD, Grubb WB, Bell JM (2002). Dissemination of new methicillin-resistant *Staphylococcus aureus* clones in the community.. J Clin Microbiol.

[33] Milheirico C, Oliveira DC, de Lencastre H (2007). Multiplex PCR strategy for subtyping the staphylococcal cassette chromosome mec type IV in methicillin-resistant *Staphylococcus aureus*: ‘SCCmec IV multiplex’.. J Antimicrob Chemother.

[34] Oliveira DC, Milheirico C, de Lencastre H (2006). Redefining a structural variant of staphylococcal cassette chromosome mec, *SCC*mec type VI.. Antimicrob Agents Chemother.

[35] Heusser R, Ender M, Berger-Bachi B, McCallum N (2007). Mosaic staphylococcal cassette chromosome mec containing two recombinase loci and a new mec complex, B2.. Antimicrob Agents Chemother.

[36] Corso A, Santos Sanches I, Aires de Sousa M, Rossi A, de Lencastre H (1998). Spread of a methicillin-resistant and multiresistant epidemic clone of *Staphylococcus aureus* in Argentina.. Microb Drug Resist.

[37] Sola C, Gribaudo G, Vindel A, Patrito L, Bocco JL (2002). Identification of a novel methicillin-resistant *Staphylococcus aureus* epidemic clone in Cordoba, Argentina, involved in nosocomial infections.. J Clin Microbiol.

[38] Orscheln RC, Hunstad DA, Fritz SA, Loughman JA, Mitchell K (2009). contribution of genetically restricted, methicillin-susceptible strains to the ongoing epidemic of community-acquired *Staphylococcus aureus* infections.. Clin Infect Dis.

[39] Tokumoto MB, Ybarra V, Torreno M, Rodriguez M, Ramirez MS (2007). Emergence of community-acquired methicillin-resistant *Staphylococcus aureus* (CA-MRSA) paediatric clone among skin and soft-tissue infections in Buenos Aires.. Int J Antimicrob Agents.

[40] Niniou I, Vourli S, Lebessi E, Foustoukou M, Vatopoulos A (2008). Clinical and molecular epidemiology of community-acquired, methicillin-resistant *Staphylococcus aureus* infections in children in central Greece.. Eur J Clin Microbiol Infect Dis.

[41] Zaoutis TE, Toltzis P, Chu J, Abrams T, Dul M (2006). Clinical and molecular epidemiology of community-acquired methicillin-resistant *Staphylococcus aureus* infections among children with risk factors for health care-associated infection: 2001–2003.. Pediatr Infect Dis J.

[42] Nubel U, Roumagnac P, Feldkamp M, Song JH, Ko KS (2008). Frequent emergence and limited geographic dispersal of methicillin-resistant *Staphylococcus aureus*.. Proc Natl Acad Sci U S A.

[43] Sola C, Saka HA, Vindel A, Bocco JL, Cordoba S. aureus Collaborative Study Group (2007). High frequency of Panton-Valentine leukocidin genes in invasive methicillin-susceptible *Staphylococcus aureus* strains and the relationship with methicillin-resistant Staphylococcus aureus in Cordoba, Argentina.. Eur J Clin Microbiol Infect Dis.

[44] Breurec S, Zriouil SB, Fall C, Boisier P, Brisse S (2011). Epidemiology of methicillin-resistant *Staphylococcus aureus* lineages in five major African towns: emergence and spread of atypical clones.. Clin Microbiol Infect.

[45] Ellington MJ, Perry C, Ganner M, Warner M, McCormick Smith I (2009). Clinical and molecular epidemiology of ciprofloxacin-susceptible MRSA encoding PVL in England and Wales.. Eur J Clin Microbiol Infect Dis.

[46] Tristan A, Bes M, Meugnier H, Lina G, Bozdogan B (2007). Global Distribution of Panton-Valentine Leukocidin-positive Methicillin-resistant *Staphylococcus aureus*, 2006.. Emerg Infect Dis.

[47] Bhattacharya D, Carleton H, Tsai CJ, Baron EJ, Perdreau-Remington F (2007). Differences in clinical and molecular characteristics of skin and soft tissue methicillin-resistant *Staphylococcus aureus* isolates between two hospitals in Northern California.. J Clin Microbiol.

[48] Rossney AS, Shore AC, Morgan PM, Fitzgibbon MM, O'connell B (2007). The Emergence and Importation of Diverse Genotypes of MRSA Harboring the Panton-Valentine Leukocidin Gene *pvl* Reveals that pvl is a Poor Marker for Community-Acquired MRSA in Ireland.. J Clin Microbiol.

[49] Monecke S, Berger-Bachi B, Coombs G, Holmes A, Kay I (2007). Comparative genomics and DNA array-based genotyping of pandemic *Staphylococcus aureus* strains encoding Panton-Valentine leukocidin.. Clin Microbiol Infect.

[50] Dauwalder O, Lina G, Durand G, Bes M, Meugnier H (2008). Epidemiology of invasive methicillin-resistant *Staphylococcus aureus* clones collected in France in 2006 and 2007.. J Clin Microbiol.

[51] Benoit SR, Estivariz C, Mogdasy C, Pedreira W, Galiana A (2008). Community Strains of Methicillin-Resistant Staphylococcus aureus as Potential Cause of Healthcare-associated Infections, Uruguay, 2002–2004.. Emerg Infect Dis.

[52] Monecke S, Ehricht R, Slickers P, Tan HL, Coombs G (2009). The molecular epidemiology and evolution of the Panton-Valentine leukocidin-positive, methicillin-resistant *Staphylococcus aureus* strain USA300 in Western Australia.. Clin Microbiol Infect.

[53] Blanco R, Tristan A, Ezpeleta G, Larsen AR, Bes M (2011). Molecular epidemiology of Panton-Valentine leukocidin-positive Staphylococcus aureus in Spain: emergence of the USA300 clone in an autochthonous population.. J Clin Microbiol.

[54] Cercenado E, Cuevas O, Marin M, Bouza E, Trincado P (2008). Community-acquired methicillin-resistant Staphylococcus aureus in Madrid, Spain: transcontinental importation and polyclonal emergence of Panton-Valentine leukocidin-positive isolates.. Diagn Microbiol Infect Dis.

[55] Sola C, Lamberghini RO, Ciarlantini M, Egea AL, Gonzalez P (2011). Heterogeneous vancomycin-intermediate susceptibility in a community-associated methicillin-resistant Staphylococcus aureus epidemic clone, in a case of Infective Endocarditis in Argentina.. Ann Clin Microbiol Antimicrob.

