# Supplementary material for: Spread of Epidemic MRSA-ST5-IV Clone Encoding PVL as a Major Cause of Community Onset Staphylococcal Infections in Argentinean Children
Source: PLoS One. 2012 Jan 23;7(1):e30487. doi: 10.1371/journal.pone.0030487 (PMC3264586; doi:10.1371/journal.pone.0030487)
Supplement: File S1 — Molecular characterization of SCCmec associated with PFGE pattern C and sequence type ST100: Pediatric clone in Argentina. (PDF) [file pone.0030487.s001.pdf]

Supplemental file S1. Molecular characterization of SCC*mec* associated with PFGE pattern C and sequence type ST100: Pediatric clone in Argentina [a]

Primer sets used for identifying SCC*mec*				SCC*mec* types				
Amplified region	Primer name	Primer sequence (5'→3')	Reference (Amplicon size, bp)	IV(2B)/ CA05 [b]	IV(2B&5)/ ZH47 ST100 Zurich [b]	V(5C2)/ WIS [b]	VI(4B)/ HDE288 [b]	IVNv/ PFGE type C ST100 Argentina [c]
ccr complex								
ccrA2	β2	ATTGCCTTGATAATAGCCITCT	32 (1000)	+	+	–	–	+
	α2	TAAAGGCATCAATGCACAAACACT						
ccrB2	ccrB2F2 F2	AGTTTCTCAGAATTCGAACG	31 (311)	+	+	–	–	+
	ccrB2F2 R2	CCGATATAGAAWGGGTTAGC						
ccrAB4	*ccrAB4 F1*	TCA TCA ATA AGT ATG GAA CG	34 (1549)	–	–	–	+	–
	ccrAB4 R1	TTT CTT GCG ACT CTC TTG G						
ccrC	ccrC F2	GTACTCGTTACAATGTTTGG	31 (449)	–	+	+	–	–
	ccrC R2	ATAATGGCTTCATGCTTACC						
Class B *mec* complex				+	–	–	+	–
IS*1272*	IS5	AACGCCACTCATAACATATGGAA	32 (2000)	+	–	–	+	–
	mA6	TATACCAAACCCGACAAC						
ψIS*1272* and *mecR1*	ISF1	AATTGAAGCAAATGCCAATCG	34 (1903)	+	–	–	+	–
	MRP1	CAACTGTCGTAGTCGAAACC						
Class B2 *mec* complex [d]				–	+	–	–	+
Tn*4001*-1 [IS1256L-aac(2')–aph(6'')]	Tn*4001*-1-F	GCCAATCGCTTAATTGGAGCCG	This study [d] (709)	–	+	–	–	+
	Tn*4001*-1-R	ACTTCATCTTCCCAAGGCTCTGT						
Tn*4001*-2 (IS*256*R-IS*1272*)	Tn*4001*-2-F	TGGCCATCACGTGTTCTGGG	This study [d] (968)	–	+	–	–	+
	Tn*4001*-2-R	TCGGATGTCTGTCCGAGGACT						
Tn*4001*-3 (*mecA*-IS256L)	Tn*4001*-3-F	ACCAAACCCGACAACTACAACTAT	This study [d] (1298)	–	+	–	–	+
	Tn*4001*-3-R	GTGTCGTAAAGCTGCGCTCA						
J3 region								
dcs	*dcs*-F2	CATCCTATGATAGCTTGGTC	31 (342)	+	–	–	+	+
	dcs-R1	CTAAATCATAGCCATGACCG						
J1 region								
SCC*mec* V	SCC*mec*V-J1-F	TTCTCCATTCTTGTTCATCC	31 (377)	–	–	+	–	–
	SCC*mec*V-J1-R	AGAGACTACTGACTTAAGTGG						
SCC*mec* IV [e]								
IVa	*J IVa F*	ATAAGAGATCGAACAGAAGC	33 (278)	+	–	–	–	–
	J IVa R	TGAAGAAATCATGCCTATCG						
IVc	*J IVc F*	CCATTGCAAATTTCTCTTCC	33 (483)	–	+	–	–	+
	J IVc R	ATAGATTCTACTGCAAGTCC						

[a] SCC*mec*: Staphylococcal Cassette Chromosome *mec*; PFGE: Pulsed field gel electrophoresis;

[b] *SCCmec* type/strain name: *SCCmec* type associated with strain control.

[c] *SCCmec* IVNv: new variant of SCC*mec* type IV associated with Pediatric clone (pulsotype C-ST100) in Argentina [15,33,34].

[d] Primers designed based on the previously determined sequence of strain ZH47 accession number AM292304 [32]. Amplicons with the expected size were end sequenced, obtaining 100% of homology with the ZH47 strain genome sequence.

[e] The SCC*mec* IV subtypes IVb, IVd, IVg, IVh also were assayed by PCR [30]**,** only positive ones are shown.
